# Evaluation of the Educational Environment at a Graduate Medical School in South Korea using the DREEM Questionnaire

**DOI:** 10.15694/mep.2019.000111.1

**Published:** 2019-05-28

**Authors:** Young Joon Ahn, Wendy Hu

**Affiliations:** 1 Department of Medical education; 2 Medical Education Unit

**Keywords:** DREEM, Medical Education, Perception

## Abstract

This article was migrated. The article was marked as recommended.

We have changed the medical educational environments which included educational curricula, medical staffs, facilities and teaching and learning methods to improve the academic achievement of medical students and to develop good doctors in schools. Medical students have complaints with their educational environments. Dundee Ready Education Environment Measure(DREEM) consists of five domains; students’ perceptions of learning, students’ perceptions of teachers, students’ academic self-perception, students’ perceptions of atmosphere. Using DREEM, the overall mean DREEM score of our school was 106.98 and low to that of Korean medical schools and developed countries. Medical students acknowledged that the domains of teachers and academic self-perceptions were positive. Through the students’ perception, the changes of the educational environment might be needed continually to get good academic achievement of medical students.

## Introduction

Professors want medical students to achieve good academic results in medicine during their campus life. However, there are many factors that influence the academic achievement of medical students. The educational environment is very important in order for medical students to accomplish good academic performance. This has been studied across the entire spectrum from primary through to tertiary level, and even beyond to postgraduate students. The educational environment is defined as everything that takes place within the classroom, department, faculty, or university and is crucial in determining the success of undergraduate medical education ( Roff and McAleerz, 2001; Genn, 2001). The educational environment encompasses student-teacher relationships, teaching and learning strategies, students’ psychosocial and emotional needs, as well as the physical structures and facilities provided by the institution ( Harden, 2001). Specifically, the World Federation for Medical Education has emphasised the learning atmosphere as one of the factors in the appraisal of medical programs ( WFME, 1998). In addition, the curriculum, the teaching-learning methods, the supporting systems and the physical environment are important factors ( Hutchinson, 2003). A productive and conductive learning environment is provided if the higher education institution is able to provide all of these.

It was helpful to increase the academic achievement of medical students and to stimulate academic motives towards improving the educational environments the students perceived and were in ( Genn, 2001; Mayya and Roff, 2004). There has been an increasing interest and concern regarding the role of the learning environment in undergraduate medical education in recent years. Students are one of the main stakeholders of the medical curriculum. Their perception provides the basis for modifying the elements in the educational environment, to enhance learning experiences in relation to teaching goals. So, to evaluate the educational environment, it is important that actions provide a high quality medical educational curriculum. It would be an important basic data to improve the educational environments.

It is important to use reliable and relevant tools to evaluate medical students’ perceptions of medical educational environments. Several tools have been developed to assess a school’s education environment over the last four decades ( Rothman *et al*., 1970; Marshall, 1978; Feletti and Clarke, 1981). The most widely used and accepted tool in medical education is the Dundee Ready Educational Environment Measure (DREEM) ( Roff *et al*., 1997; Roff, 2005), developed by Roff *et al* ( Byrnes *et al*., 2012). The DREEM is a 50-item measure of students’ perceptions of their learning environment, with five scales recording the perception of learning, perception of teachers, academic self-perception, perception of atmosphere and social self-perception. It has been used for many purposes, like identifying the strengths and weaknesses of a teaching program, comparing the outcomes of a program delivered at different centres, assessing the academic achievement of medical students and evaluating changes after improving the medical educational curriculum ( Mayya and Roff, 2004; Jiffry *et al*., 2005; Roff *et al*., 2001; Al-Hazimi *et al*., 2004; Zawawi and Elzubeir, 2012).

There have been many studies on educational environments in many countries. However, there have been few studies conducted in South Korea. Over the past two decades, there have been remarkable changes and developments in medical education in South Korea. Efforts have been made to introduce various supporting educational systems and new medical curricula from developed countries, to improve the academic achievements of medical students. According to new trends in Korean medical education, medical schools developed new curricula, such as PBL (problem based learning), PDS (patient-doctor-society relationship), integrated subject course, OSCE (objective structured clinical examination) and CPX (clinical performance examination).

The medical education environment in this school will be investigated. It is necessary to objectively examine students’ awareness of the current medical education environment in one school in South Korea and compare this with other countries. We will provide basic data to improve the medical environment and to develop a new curriculum for South Korea.

## Methods

### 1. Participants

This study included first year to fourth year students at one graduate school of medicine, located far from the capital of South Korea. Information was collected through the DREEM questionnaire, as well as on demographic characteristics. 464 out of 500 medical students took part (response 92.8%).

### 2. Measures

#### 1) Dundee Ready Educational Environment Measure

The DREEM questionnaire, as well as several questions on demographic characteristics (age, gender, year of study, religion, major), was self-administered to all eligible students. The DREEM questionnaire was used as a measure of students’ perceptions of the educational environment. The DREEM is a 50-item inventory, involving statements related to the educational environment, with five domains and a maximum score of 200. The first domain is students’ perceptions of learning (SPL), which contains 12 items and has a maximum score of 48. The second is students’ perceptions of teachers (SPT), which contains 11 items, with a maximum score of 44. The third is students’ academic self-perceptions (SASP), which contains eight items, with a maximum score of 32. The fourth is students’ perceptions of atmosphere (SPA), which contains 12 items, with a maximum score of 48. The fifth is students’ social self-perceptions (SSSP), which contains seven items, with a maximum score of 28.

The participating students were provided with an explanation of the study’s purpose and the DREEM questionnaire, as well as a consent form. Students were asked to read each statement carefully and to respond using a five-point Likert-type scale, ranging from strongly agree (4) to strongly disagree (0). Incomplete questionnaires were excluded from the study. Reverse scoring was used for the nine negative items (4, 8, 9, 17, 25, 35, 39, 48, and 50) where strongly agree was scored 0 and strongly disagree scored 4. The McAleer and Roff practical guideline [4] was used to interpret the results. An overall score of 0 to 50 was very poor, 51 to 100 indicated plenty of problems, 101 to 150 indicated more positive than negative, and 151 to 200 was excellent. The DREEM items with a mean score of ≥3.5 indicated positive points, ≤2 indicated problem areas, and between 2 and 3 indicated aspects of the environment that could be improved [17].

#### 2) Statistical analysis

The data was compiled in an Excel spreadsheet. Means and standard deviations were calculated. Total scores and the domain scores for all students were calculated. Data was analysed using the statistical package SPSS, version 20.0 (IBM Corp., Armonk, USA). One-way analysis of variance (ANOVA), with a post hoc Tukey-Kramer multiple comparisons test. Data are presented as mean ± standard deviation. Statistical significance was present as a) p<0.05, b) p<0.01 and c) p<0.001 and was used to identify the significant differences between subgroups. A p-value of less than 0.05 was considered significant. The Cronbach alpha was 0.94. The validity of the subscale items ranged from 0.66 to 0.85.

#### 3) Academic achievement

We confirmed the students’ academic performance, measured by self-report evaluation, in response to the student-rated academic performance. We classified the results of the previous semester’s academic performance into upper, middle high, middle, middle lower and lower and analysed it.

### 3. Data collection and analysis

The data collection of this study was conducted from November to December in 2016, by a questionnaire survey of students from a local medical school. First, we explained the contents of the questionnaire and write the answers. The questionnaires were conducted in the same group of grades. The medical students took about 20 minutes to complete the questionnaire. The results were analysed as follows. First, the mean and standard deviation were obtained, to examine the distribution of DREEM scores. Second, a one-way ANOVA was conducted to examine differences in DREEM according to gender, grade and academic achievement. Third, the mean and standard deviation of each DREEM item were calculated.

## Results/Analysis

### 1. Demographic data

A total of 464 students out of 500 (92.8%) responded to the questionnaires, from first grade to fourth at a local graduate medical school. There were 239 male students (51.5%) and 225 female students (48.5%). 105 students (22.6%) were in the first grade, 112 students (24.2%) in the second grade, 122 students (26.3 %) in the third grade and 125 students (26.9%) in the fourth grade. There were 196 atheists (42.2%), 138 Christians (29.7%), 84 Catholics (18.1%) and 46 Buddhists (10.0%). There were 281 (60.6%) over 28-year-olds, 146 (31.5%) from 24 to 27-years-old, and 37 (7.9%) younger than 23-years-old. By major, there were 137 in engineering (29.5%), 171 in basic science (36.9%), 58 in humanities and society (12.5%), 50 in public health (10.8%), 9 in arts and gym (1.9%) and 39 in other (8.4%; Table 1).

**Table 1.** Demographic Characteristics of Subjects

Variable	Response	N	%
Gender	Male	239	51.5
	Female	225	48.5
Grade	1st year	105	22.6
	2nd year	112	24.2
	3rd year	122	26.3
	4th year	125	26.9
Religion	Atheist	196	42.2
	Christian	138	29.7
	Catholic	84	18.1
	Buddhist	46	10.0
Age	Younger than 23	37	7.9
	From 24 to 27	146	31.5
	Older than 28	281	60.6
Major	Technical Engineering	137	29.5
	Basic Science	171	36.9
	Humanities and Society	58	12.5
	Public Health	50	10.8
	Gym and Arts	9	1.9
	Other	39	8.4
Total		464	100.0

### 2. The average DREEM score and subscales

Table 2 shows the subscales and total DREEM mean and percentage scores. The mean score of the DREEM was 106.98 out of 200 (53.49%), and the mean scores of each item were 24.80 (51.60%) for SPL, 25.67 (54.98%) for SPT, 16.97 (57.01%) for SAS, 24.82 (51.93%) for SPA and 14.72 (53.07%) for SSS. The average score of the DREEM was 53.49 out of 100 and the average scores of each subscale were 51.60 for SPL, 54.98 for SPT, 57.01 for SAS, 51.93 for SPA and 53.07 for SSS. The highest score was for the SAS subscale and the lowest score was for the SPL subscale.

**Table 2.** Subscales and Total DREEM Mean and Percentage Scores (n=464)

DREEM Subscale	N	Mean	SD	%
SPL	464	24.80	±6.31	51.60
SPT	464	25.67	±4.98	54.98
SAS	464	16.97	±4.24	57.01
SPA	464	24.82	±5.99	51.93
SSS	464	14.72	±3.7	53.07
Total score		106.98	±18.73	53.49

### 3. Score distribution of DREEM for each characteristic

DREEM scores were highest for students in their senior year, lowest for students in their first year and were significantly different for fourth year and third year students, compared to first year students (p <0.05). DREEM scores were significantly higher in males than in females (p <0.05). Academic achievement was divided into upper, middle upper, middle, middle lower and lower grades. DREEM scores of the students who thought they had high academic achievement were significantly higher than those of the students who thought they had lower academic achievement (p <0.05). In other words, the higher the academic achievement, the more positive the perception of the medical education environment. There were no significant differences in DREEM scores by religion or club activities( Table 3).

**Table 3.** DREEM Scores by Characteristics

Variable	Responses	N	Score (Average ± SD)	P value
Year	1	105	108.68±17.71	0.009
	2	112	101.90±17.34	
	3	122	111.30±19.00	
	4	125	105.90±19.47	
Sex	Male	239	109.37±19.26	0.023
	Female	225	103.69±18.08	
Age (years)	Younger than 23	37	111.00±19.22	0.087
	24 to 27	146	104.53±18.49	
	Older than 28	281	106.93±19.31	
Religion	Atheist	196	106.91±19.47	0.59
	Theist	268	106.65±17.81	
Club activity	Yes	365	106.80±18.73	0.86
	No	99	105.67±17.32	
Achievement	Higher	90	111.49±16.33	0.015
	Middle higher	121	108.29±15.59	
	Middle	134	105.27±14.67	
	Middle lower	77	105.66±14.55	
	Lower	42	103.30±13.82	
Total		464		

### 4. DREEM score of each item

We found items with a mean score of less than two and more than three ( Table 4). In the SPL subscale, items with a mean score of less than two were questions 13 (M ± SD = 1.57 ± .85), 25 (M ± SD = 1.81 ± .92), 44 (M ± SD = 1.98 ±. 77) and 48 (M ± SD = 1.65 ± .78). In the SPT subscale, items with a mean score of less than two were questions 9 (M ± SD = 1.52 ± .89), 29 (M ± SD = 1.95 ± .78) and 39 (M ± SD = 1.50 ± .84). In the SAS subscale, items with a mean score of less than two were questions 5 (M ± SD = 1.80 ± 1.05) and 27 (M ± SD = 1.41 ± .92). In the SPA subscale, items with a mean score of less than two were questions 11 (M ± SD = 1.74 ± .78), 12 (M ± SD = 1.93 ± .88), 35 (M ± SD = 1.47 ± .82), 42 (M ± SD = 1.96 ± .87) and 49 (M ± SD = 1.73 ± .90). In the SSS subscale, items with a mean score of less than two were questions 3 (M ± SD = 1.20 ± .90), 14 (M ± SD = 1.95 ± .92) and 28 (M ± SD = 1.90 ± .87). The score for question 3 (M ± SD = 1.20 ± .90) was the lowest. The item with a mean score of more than three was question 2 (M ± SD = 3.00 ± .65) in the SPT subscale and was the highest of the 50 items.

**Table 4.** DREEM Items Mean Scores (n=464)

Items	N	M	SD
SPL			
1. I am encouraged to participate in class	464	2.59	0.78
7. The teaching is often stimulating	464	2.06	0.81
13. The teaching is student-centred ^*^	464	1.57	0.85
16. The teaching helps to develop my competence	464	2.41	0.76
20. The teaching is well focused	464	2.33	0.70
22. The teaching helps to develop my confidence	464	2.06	0.78
24. The teaching time is put to good use	464	2.06	0.82
25. The teaching over-emphasizes factual learning ^*^	464	1.78	0.83
38. I am clear about the learning objectives of the course	464	2.20	0.66
44. The teaching encourages me to be an active learner ^*^	464	1.98	0.77
47. Long-term learning is emphasized over short-term learning	464	2.04	0.94
48. The teaching is too teacher-centred ^*^	464	1.65	0.78
SPT			
2. The teachers are knowledgeable	464	3.00	0.65
6. The teachers are patient with patients	464	2.44	0.72
8. The teachers ridicule the students	464	2.14	0.82
9. The teachers are authoritarian ^*^	464	1.52	0.89
18. The teachers have good communications skills with patients	464	2.36	0.70
29. The teachers are good at providing feedback to students ^*^	464	1.95	0.78
32. The teachers provide constructive criticism here	464	2.33	0.74
37. The teachers give clear examples	464	2.35	0.70
39. The teachers get angry in class ^*^	464	1.50	0.84
40. The teachers are well prepared for their classes	464	2.50	0.70
50. The students irritate the teachers	464	2.09	0.76
SAS			
5. Learning strategies that worked for me before continue to work for me now ^*^	464	1.66	1.02
10. I am confident about my passing this year	464	2.82	0.84
21. I feel I am being well prepared for my profession	464	2.26	0.73
26. Last year’s work has been a good preparation for this year’s work	464	2.45	0.74
27. I am able to memorize all I need ^*^	464	1.41	0.82
31. I have learned a lot about empathy in my profession	464	2.57	0.66
41. My problem-solving skills are being well developed here	464	2.35	0.67
45. Much of what I have to learn seems relevant to a career in healthcare	464	2.72	0.71
SPA			
11. The atmosphere is relaxed during the ward teaching	464	1.74	0.78
12. This school is well time-tabled	464	1.93	0.88
17. Cheating is a problem in this school ^*^	464	2.72	1.11
23. The atmosphere is relaxed during lectures	464	2.15	0.75
30.There are opportunities for me to develop interpersonal skills	464	2.19	0.92
33. I feel comfortable in class socially	464	2.31	0.74
34. The atmosphere is relaxed during seminars/tutorials	464	2.36	0.73
35. I find the experience disappointing ^*^	464	1.47	0.82
36. I am able to concentrate well	464	2.21	0.67
42. The enjoyment outweighs the stress of the course	464	1.96	0.87
43. The atmosphere motivates me as a learner	464	2.18	0.73
49. I feel able to ask the questions I want	464	1.73	0.90
SSS			
3. There is a good support system for students who get stressed	464	1.20	0.85
4. I am too tired to enjoy the course ^*^	464	2.01	0.83
14. I am rarely bored on this course	464	1.95	0.92
15. I have good friends in this school	464	2.97	0.68
19. My social life is good	464	2.71	0.66
28. I seldom feel lonely	464	1.90	0.87
46. My accommodation is pleasant	464	2.09	0.81

* *Negative item: low score indicates agreement*

## Discussion

The educational environment includes all the physical, psychological and social contexts in which learners interact to achieve the educational curriculum ( Roff, 2001). Assessing the educational environment is a key activity in providing high quality medical educational courses. Student feedback on the educational environment is the most important evidence for improving the educational environment ( Genn, 2001).

The purpose of this study was to investigate the perceptions of medical students from one medical school in South Korea on their educational environment, using DREEM. The score distributions were examined by item, age, gender and the level of academic achievement. The results are summarised and discussed below.

The average overall score of the DREEM was 106.98 out of 200 (53.5%). This was within the positive category of 101 to 150. The highest score was 127 and the lowest score was 88. Previous studies have produced DREEM scores above 130 in European universities, such as the UK’s Dundee Medical School (Al-Hazimi *et al*., 2012). The DREEM score of the medical school in this study was below the mean 113.76 of 40 Korean medical schools ( Kwi *et al*., 2015) and was similar to those of Sri Lanka ( Jiffry *et al*., 2005), Nigeria ( Roff *et al*., 2001) and India ( Mayya and Roff, 2004; Kohli and Dhaliwal, 2013). Among 40 South Korean medical schools, the DREEM mean score of the school was lower than that of other schools and they perceived it negatively because their school was located far from the capital of Korea.

In the results of previous studies, many universities have increased their scores through student centred-education, an integrated curriculum and problem-based learning, according to the trends of medical education since the 20 ^th^ century. In Korea, many universities have changed their medical curriculum to improve their scores (KJME,2014). But even though they had many attempts to improve the scores, the students’ perception of the medical education environment was not improved and so we need inward changes of medical environment. The average DREEM scores of the medical school in this study were lower than those of other medical schools in Korea, except for facilities and educational content, because this medical school was far from the capital city. The DREEM score in the SAS subscale was higher than the other subscales. This is different from the Korean national survey, which had higher scores in the SPT subscale.

For each item, we examined 17 items with an average score of two or less, which were interpreted as problematic areas by standard criteria. Items with less than two points were more common in the SPA subscale. In the SPL subscale, the students recognised that the lessons were not student-centred (item 13), were too professor-centred (item 48) and factor-centred (item 25) and that they were not encouraged (item 44). This implies that the lessons were still professor-centred and did not encourage the students to be active learners, which is similar to the other medical schools in South Korea ( Kwi *et al*., 2015). In previous studies, universities using the lecture-centred traditional curriculum have also shown these problems ( Zawawi and Elzubeir, 2012;KJME, 2014).

The officers of most medical schools in Korea have emphasised a learner-centred curriculum. The integrated curriculum, and various teaching-learning methods such as PBL and TBL, have spread widely and made every effort to establish a suitable educational environment. And then many things were changed a lot ( Kohli and Dhaliwal, 2013). However, in spite of the continuous improvement of medical education and the efforts towards change, it is significant that medical students did not perceive a change in the problems with the traditional curriculum.

In the SPT subscale, the students recognised that the professors were knowledgeable, with an average score of more than three points, but felt they were also authoritarian (item 9), easily angered (item 39) and gave poor feedback (item 29). Students feeling that professors are authoritarian has not changed and is one of the most difficult parts of the educational environment ( Genn, 2001; Kohli and Dhaliwal, 2013; Brown, Williams and Lynch, 2011). University governors must provide faculty development programs to improve teachers’ communication with students and to develop teaching-learning methods suitable to the new trends in medical educational and educational environments.

In the SAS subscale, the students perceived that they had problems with learning strategies (item 5) and memory of medicine because they had a lot of their major (item 27). In the SPA and SSS subscales, the students recognised that they were stressed (item 42), felt lonely (item 28), were sometimes bored (item 14) and lacked a good support system. They gave the lowest score for the question relating to a good support system for stressed students. Now, efforts need to be made to ensure there is a good support system for the learning and welfare of students. Particularly, a good support system for students who have experienced failure or low academic achievement is needed. We examined only one item with an average score of three. The students felt that the teachers were knowledgeable (item 2) and this score was the highest.

The DREEM scores for each item were significantly different for different student demographics. The DREEM score of second year students was significantly lower than that of any other year. The DREEM score of fourth year students was also low. These results were similar to the Korean national survey and other countries. This may be because second year students have many clinical subjects and fourth year students are stressed about Korean medical licensing examinations; therefore, perceiving the medical educational environment negatively. The DREEM score for male students was significantly higher than for female students. The articles in three universities of the Middle East which have run traditional teacher-centred curriculum showed that DREEM score in male was higher than in female and DREEM score was analogous between them in Dundee which has run student-centred curriculum. The results of this study were contrary to research demonstrating that female students perceive the educational environment as friendlier and more positive in various studies ( Al-Hazimi *et al*., 2004; Dasputra, Chari and Gade, 2014). In addition, some studies have reported that there was no significant difference in DREEM scores according to sex ( Al-Hazimi *et al*., 2004; Dasputra, Chari and Gade, 2014; Miles and Leinster, 2007).

The DREEM score of students who had higher academic achievement was higher than the scores of students who had middle or lower academic achievement. This result was similar to the Korean national survey and other countries ( Roff *et al*., 2001). Students with higher academic achievement were more self-directed, they were at the level of adult learners and perceived the medical educational environment to be positive and student-centred, compared to students with lower academic achievement. On the other hand, students with low achievement were not satisfied with the medical educational environment ( Brown, Williams and Lynch, 2011). As there was obviously a difference in the perception of the medical educational environment according to achievement level, educational support for students with low achievement is needed. However, DREEM scores were not different significantly according to age, religion and club activities. Usually older, more mature graduate students accept the medical educational environment positively ( McAleer and Roff, 2001).

In this research, there were some limitations. Although the level of academic achievement in this study was an important variable in the students’ perception of the medical education environment, this was limited, because it was based on the students’ self-rated responses, rather than actual grades. Even if professionals have undergone reviewal and modified process of questionnaire, there were limitation that could not be completely eliminated regarding uncertainties and ambiguous meanings in items. Even with the limitations, this study investigated students’ perceptions of a medical educational environment and provides evidence for improving the educational environment. Further study on the educational environment, not only for medical students, but also public health students and clinical residents is needed.

## Conclusion

Students’ perceptions of their educational environment of our graduate medical school in South Korea were negative in many aspects, compared to other schools. Even though the school had developed student-centred curricula and facilities, there were differences between students’ perceptions and the school’s ideal. Students’ perceptions need to be investigated periodically to improve the educational environment in conjunction with the students.

## Take Home Messages

• The assessment of educational environment is required periodically.
• Medical curriculum will be improved by reflections on the thought of the students’ perceptions.
• The bad perceptions of students can influence the achievement, negatively.

## Notes On Contributors

Dr. Ahn is a Professor, Director of the Department of Medical Education, College of Medicine, Chosun University.

Dr. Hu is a Professor, Associate Dean of Medical Education Unit, School of Medicine, Western Sydney University, Campbelltown, New South Wales, Australia.
