# Supplementary material for: Evaluation of the Educational Environment at a Graduate Medical School in South Korea using the DREEM Questionnaire
Source: MedEdPublish (2016). 2019 May 28;8:111. [Version 1] doi: 10.15694/mep.2019.000111.1 (PMC10712468; doi:10.15694/mep.2019.000111.1)
Supplement: Peer review history

# Reviewer report: Reviewer response for version 1

**DOI:** 10.21956/mep.19804.r29799

**Published:** 2019-09-19

**Authors:** Ken Masters

**Affiliations:** 1 Sultan Qaboos University

This review has been migrated. The reviewer awarded 3 stars out of 5 An interesting paper on evaluating educational environment at a graduate medical school in South Korea using the DREEM questionnaire. An interesting paper, although it does require work:• The Abstract should be laid out with the same sub-headings as the main headings of the paper, so that the reader can more easily get a sense of the paper. • In the Introduction, there are several statements that require support from the literature, as it is difficult to distinguish from the authors’ own opinions and what has already been established in the literature.• Unless there is good reason (which should be then stated), there is no need to attempt to hide the name of the school, and it should be stated clearly in the paper.• Given that there were result differences between the years of study, it would be a good idea if authors could give some details of the curriculum, especially the level of clinical exposure during the degree.• There is some repetition about the DREEM questionnaire construction in the Introduction and the Methods, and this should be removed.• In Table 2, given that N=464 for all, and is stated in the Table caption, the column showing it can be removed.• The text in the paragraph before Table 2 repeats what is in Table 2. Please remove most (all?) of that paragraph.• Similarly for Table 4 – please remove the N column from the Table, and all repetition from the paragraph (a brief commentary on the Table is fine, but not repeating so much of the data).• The Conclusion is really thin, and undermines the value of the research. The authors should do themselves a favour and expand somewhat on the Conclusion.• The Take-Home messages also require some work. These are supposed to represent the most important findings of the research. Number 1 and Number 2 may be true, but do not appear to be a finding of the research. Number 3 assumes a one-way causal relationship that has not been established in the study. (Does happiness cause higher achievement, or does higher achievement cause happiness? There is an association, but no causal relationship)Minor issues:• There are some small language errors that could be corrected with a closer proof-read of the paper. Also, “data was” should be “data were”.• Try not to start sentences with a number (E.g. “105 students…”)So, overall, an interesting paper, but the authors need to correct some of the work, especially supplying some more detail in some area, and reducing the repetition in the Results.

Reviewer Expertise:

NA

No decision status is available

# Reviewer report: Reviewer response for version 1

**DOI:** 10.21956/mep.19804.r29798

**Published:** 2019-06-24

**Authors:** Paolo Colet

**Affiliations:** 1 Nazarbayev University

This review has been migrated. The reviewer awarded 4 stars out of 5 This is a very good study. The introduction to the conclusion is well thought. One thing I like is, it does compare with international studies, especially in the introduction and discussion sections. However, a few things need to be edited, like the objective of the study on the introduction part, which should be in the past tense. On the statistical analysis, I am not sure if the reported Cronbach alpha is from the previous study or the current study. On the Results section, it seems to me a table reading. In addition, when beginning a sentence with a number, it needs to be spelled out. Overall, this study is significant on its own as we move toward improving our medical education.

Reviewer Expertise:

NA

No decision status is available

# Reviewer report: Reviewer response for version 1

**DOI:** 10.21956/mep.19804.r29800

**Published:** 2019-06-24

**Authors:** Hyoseon Choi

**Affiliations:** 1 Chosun University College of Medicine

This review has been migrated. The reviewer awarded 5 stars out of 5 The medical education environment has complexity and uncertainty. It is of great significance to have conducted leading studies on the medical education environment. I think it is a well-structured study from a clear research problem to a conclusion. I was deeply impressed with the discussion. I think it will be a better paper after proof reading.

Reviewer Expertise:

NA

No decision status is available

# Reviewer report: Reviewer response for version 1

**DOI:** 10.21956/mep.19804.r29797

**Published:** 2019-06-24

**Authors:** Seongpyo Mun

**Affiliations:** 1 Department of surgery, school of medicine, Chosun University, Gwangju, Republic of Korea

This review has been migrated. The reviewer awarded 4 stars out of 5 The results are very impressive and educational. It would be more easier for the reader to understand the results if you provide the details of questionnaire as appendices. You conclude that some low scoring items is caused by geographic location of the school without providng the support. I recommend you to provide the evidence that support your insist on this conclusion. This study make us remind the importance of educational environment.

Reviewer Expertise:

NA

No decision status is available
